# AI product liability under EU and Canadian laws

**DOI:** 10.3389/frai.2026.1709945

**Published:** 2026-06-24

**Authors:** Jawahitha Sarabdeen

**Affiliations:** College of Law, Prince Sultan University, Riyadh, Saudi Arabia

**Keywords:** AI products, Canada, EU, laws, strict liability, tort liability

## Abstract

AI and AI-enabled product-related liability have been a concern for the industries and consumers. They would like clarity on the liability for defective AI systems or products to mitigate risks. Laws in many countries provide tort or strict liability to address product liability. Some countries are amending or seeking to amend existing product liability legislation to address new issues related to AI. The EU has passed new directives and amended existing ones to address AI product liability, while Canada has drafted a new law to address this issue. The article aimed to assess the available literature and identify gaps in AI product liability. It also investigates the rules in the EU and Canada regarding AI product liability. By doing so, it intended to suggest improvements to the Canadian law on AI product liability. To achieve the objective, the researcher utilized a systematic literature review, legal doctrinal research, and comparative legal method. The finding showed that proving fault in an AI environment is challenging. Therefore, strict liability or a rebuttable presumption of causation could effectively protect end users. It will also ensure that various parties involved in AI take necessary safety measures to meet the legal presumption of their liability. It further revealed that the EU regulatory framework addresses AI product liability more effectively, although it has a few weaknesses that need to be addressed. The article suggested improvements to Canadian law regarding proof of fault, personal remedies, the role of the court, and the parties’ liability.

## 1 Introduction

AI products or AI-enabled products are available in the market. As the use of these products increases, the question of product liability becomes a debated topic. Currently, many countries address product liability through existing legislation. The current fault-based tort liability regime for AI product-related claims will leave victims of AI-related harm with a lower level of protection than for other products and services. The applicability of tort liability is difficult as there is a blurred line of responsibility of various parties in AI system design, development, and implementation. Identifying a flaw in a complex system with updates from multiple parties requires detailed expert input to ensure liability. Automation has created further difficulties as human players have become invisible and distant.

The EU passed directives and regulations to address various issues posed by AI (Mügge, 2024). It passed the Artificial Intelligence Act (AIA) and revised the Product Liability Directive to address AI liability and standardize law on AI (Minssen et al., 2024). The standardization of the law can help ensure it is applied and interpreted effectively. The AIA defined AI as software developed using one or more techniques specified in the law that produces a defined outcome. The definition is technologically neutral. AIA explained the high-risk technology as a product that can harm health, safety, and fundamental rights. The assessment of AI risk should investigate the purpose; the extent or likely use; the harm; the victims’ dependency on the system and their vulnerability; the reversibility of the harmful outcome; and the available redress mechanisms. Tort or product liability may arise when AI systems cause or facilitate harm due to a system error or its defective output.

The AI products are different from traditional products. For example, the AI software is intangible, so it may not be as clear as other products. The liability law, which covers tangible goods, may apply to defective design, process, or ineffective warning. The new EU Product Liability and AIA address this issue. Further, AI software is not installed once; it requires occasional updates. Does the product liability apply to the original placing of the AI product, or does it cover updated products? Who is liable for system or design errors in an AI ecosystem is not clearly articulated in the law. The literature is also scattered on this issue (Çela et al., 2025).

Canada, following the EU development, drafted the Artificial Intelligence and Data Act (AIDA) to address high-impact AI systems; unfortunately, the law died before it was passed. The AIDA covered AI design, development, and deployment for trade and commerce. It is intended to cover AI products that are placed in the market (Harvey and Gill, 2025). Despite the demise of AIDA, Canadian government initiatives on safe AI governance that balance innovation with ethics can be seen in the 2017 national AI strategy and the Global Partnership on AI. To build a competitive AI industry, various AI tools were introduced at all levels of government (federal, provincial, and sectoral). Treasury Board policy instruments could also help clarify the AI policy, standards, directives, and guidelines for federal offices. Failure by the offices to follow those instruments will result in various consequences, including budget restrictions. These measures are not robust oversight of government activities, but can be considered as a controlling mechanism in the absence of laws and regulations. Three of the tools are important to regulate AI use by federal government entities.

1. Directive on Automated Decision-Making: This is applied to automated decision-making to ensure transparency and control risk.
2. Algorithmic Impact Assessment Tool: Used to understand and assess the impact of automated decision-making.
3. List of Interested AI Suppliers: It provides a list of pre-qualified suppliers for effective AI use by federal government entities.

At the provincial level, legislation is also crucial, as many AI-impacting industries fall under provincial jurisdiction. In this regard, Ontario introduced Bill 194, which requires the provincial public sector, including education institutions, law enforcement, hospitals, and others, to comply with AI-related obligations. The public sector is expected to implement rules related to AI use, accountability, transparency, and risk management. Similarly, British Columbia introduced draft AI responsible use principles, and Quebec’s Ministère de la Cybersécurité et du Numérique (MCN) has issued responsible use guidelines for public bodies. Additionally, the Quebec Civil Code added extra-contractual liability that can impose liability on manufacturers. Though federal regulation is important in Canadian AI governance, as it establishes centralized oversight across the country, existing laws, measures, directives, and tools may address some concerns about AI. For example, the algorithm bias could be covered by The Canadian Charter of Rights and Freedoms or The Canadian Human Rights Act.

Additionally, provincial legislation could help fill some gaps in AI governance. Future comprehensive federal legislation could help address AI risks throughout its product life cycle. Currently, for AI product liability, the claim should be brought under tort of negligence.

Hence, the article aims to analyze the EU directive and regulation on AI product liability and the tort liability regime in Canada to understand the AI product liability regime. The analysis will examine the current product liability regimes in the EU and Canada and identify weaknesses in the current law. The comparative lens highlights how countries conceptualize responsibility in AI governance and how they approach it. The analysis will lead to suggestions for improving AI product liability laws for AI design and beyond (Bradford v. GlaxoSmithKline LLC et al. (2023), U.S. District Courts, case no. 3:23-cv-16983). In addition, there is a lack of research on AI product liability; this study will fill the gap in the literature.

## 2 Literature review

### 2.1 Regulation of AI

The literature on AI and product liability is scattered and underdeveloped. There is little literature on AI product liability. Generally, the literature on AI and the law focuses on issues such as data privacy, accountability, explainability, ethics, regulation, copyright, and transparency rather than on product liability. Çela et al. (2025) discussed the use of AI by legal professionals and the related problems. The authors wrote that AI systems are used for administrative, document review, and compliance purposes. The courts also use autonomous AI systems, such as COMPAS (i.e., Correctional Offender Management Profiling for Alternative Sanctions), to aid decision-making. The AI used by lawyers and courts is considered high risk under AIA, as it poses potential liability, transparency, and explainability issues that warrant regulation.

Transparency in AI technologies has narrowed to explainability and the transparency requirement under AIA. Though informed and critical analysis of the transparency provisions can create opportunities for responsible AI, the provisions in AIA allowing secrecy could enable avoiding accountability. To avoid this issue, it is necessary to maintain transparency as a core principle and prohibit secrecy (Busuioc et al., 2023). The use of technology for traceability of information to consumers cannot succeed if ethical and privacy issues are not addressed (Chrysochou et al., 2009). The current EU regulation on AI is full of cracks in its facade. The loopholes can be noticeable in the EU member states’ constitutions, in the treaties, charters, regulations, etc. As such, the current legal framework in regulating AI should be reconsidered (Kuch, 2023). Gupta (2025) suggested removing regulations on the legal profession to enable large-scale legal AI to provide legal services. This can remove barriers to getting legal services and promote access to justice. Legal AI might create accountability and avoid bias. Any issues caused by Legl AI can be controlled through technology and regulatory solutions.

On copyright law and AI, Peukert and Windisch (2025) discussed the importance of copyright and of enabling creative reuse, new licensing, and business models. They also looked at the possibility of algorithmic and software licensing. It is argued that the conventional ‘protectionist’ approach and the non-conventional ‘exclusionary’ rights-privileged approach may not be relevant to the use of AI. One new approach could be to introduce property into liability. This will ensure fair compensation for copyright owners for the use of their work by AI (Ghidini, 2025). Tammenlehto and Kallio (2025) investigated the allocation of criminal intent when part of a copyright violation is committed using an AI system, as the current legal framework may not provide the right solution. They suggested exploring alternatives to the current determination of criminal liability.

Gerke and Simon (2025) investigated the use of AI in the medical field and product liability. They considered the recent USA case in Louisiana (Dickson v. Dexcom Inc., 2024, 2:24-CV-00121) regarding an enabled medical device and the manufacturer’s liability. The main question was whether manufacturers are liable for injuries caused by authorized products. The literature also showed that AI-enabled New Product Development (NPD) revolutionized NPD, helping with decision-making, reducing time, and providing market insights. However, the development of NPD can be negatively affected if regulatory compliance is not clearly stated (Mubarak et al., 2024).

The research also examined the integration of artificial intelligence (AI) into healthcare. The study suggested drafting a framework for responsible AI that involves all stakeholders and clearly demarcating the parties’ liabilities (Bouhouita-Guermech and Haidar, 2024). Liability demarcation among various stakeholders has also been considered in a study on advancements in neurotechnology. The AI technology can improve the diagnosis and treatment of mental illness. However, a few challenges could disrupt development and application. One of the major challenges is allocating liability among the parties. The study suggested introducing measures to address neuro-rights, protect data privacy and security, address bias, and provide guidelines for the distribution of the technology, as it has the potential to alter humans (Goering et al., 2021). The public value approach, as a multidimensional concept, can be used to tailor policies to different beneficiaries comprehensively. This can promote wider AI governance and adoption (Lewerenz et al., 2025).

Bélisle-Pipon and Victor (2024) argued that AI system developers and the regulators dump the responsibilities on users and hosts who are not responsible for the AI. They found that AI ethics formulated by system developers and regulators are broad and unenforceable. They promote institutional implementation without understanding the feasibility. The ethical guidelines are disconnected from practice. To avoid ethics dumping, there is a need to empower users, enhance stakeholder engagement, accountability, and contextualize ethical guidelines. The guidelines should identify responsible parties, introduce incentives, provide trusted resources, ensure clear communication, and enforce measures to improve policy and legislative posture (Hoong and Rezania, 2024).

### 2.2 Product liability for an AI product

Regarding product liability, the literature showed inconsistent laws even among common law countries. Some laws define “product” as tangible. Others have taken a broader definition that includes software and AI systems within the product. The EU Product Liability Directive includes AI as a product and imposes strict liability for defects. This approach differs from the USA (Lubin, 2025). The new EU Directive on Product Liability widens the definition and imposes continued post-marketing control by the manufacturer. The protection also extends to data used for private purposes. The liability applies to all products, including digital products or elements. The liability is imposed to cover broader stakeholders: e-commerce intermediaries, manufacturers, importers, and distributors (Wagner, 2024). The changes in technology and business models, the occurrence of harm, and the involvement of third parties require expanding manufacturers’ or commercial intermediaries’ liability; the new product liability directive addresses this (Machnikowski, 2024).

For AI product liability, writers are divided on its application. Some suggested tort of negligence or fault-based liability, while others proposed strict liability or strict product liability. Some argue that strict liability will be a better option for protecting consumers from defects in AI products, given their inherent characteristics of opacity, connectivity, data dependency, autonomy, and self-learning. These characteristics are expected to make it difficult to identify or trace a potential problem. Changes in technology prompted changes to workers’ compensation statutes and led to the adoption of no-fault automobile insurance systems or a “strict” products liability regime in the USA. The changes were made after considering factors such as the frequency of severe harm, the difficulty of proving harm, and the social benefits of the technology. The development of the AI technological revolution may lead to the application of strict product liability to relieve victims of the need to prove a complex web of technological errors (Gifford, 2018). Yuanitasari et al. (2023) also suggested applying strict liability rather than fault liability to protect consumers of AI products. Strict products liability was introduced to address products that did not meet consumers’ ordinary expectations, or that malfunctioned and violated the implied warranty of quality (Geistfeld, 2021).

The literature on negligence liability suggests that negligence liability is preferable to strict liability in cases involving autonomous computers, robots, or machines. When negligence law is applied to an autonomous computer system, it can be treated as a computer tortfeasor rather than a product. This could help to enhance safety features (Abbott, 2018). In cases of data breaches, tort liability should apply rather than strict liability. In cases of a data breach involving a data controller, the care could increase. If the loss from a data breach is lower, the care level could be reduced. Thus, negligence could address data breaches and their subsequent damages more effectively than strict liability (Jun and Kim, 2024; Chamberlain, 2023; and Kellam and Nottage, 2008).

### 2.3 Future direction on product liability on AI products

While the debate over the application of negligent liability or strict liability continues, some researchers suggest a different approach to AI product liability. The combination of AI, products, and machines disrupted manufacturing processes that involve humans at every stage. Specifically, the absence of agency or personhood raises a new liability question. Some product behaviors cannot be predicted and explained. Therefore, introducing a supplementary rule alongside the existing liability rules could regulate AI product liability. The supplementary rules can be set up as quasi-safe harbors or predetermined levels of care. Once the predetermined level is met, the case can fall within current tort liability. Failure to prove that level can lead to strict liability. The predetermined rules can relate to design, monitoring duties, built-in emergency systems, continuous support, and related duties. The design can incorporate fail-safes, error-correction measures, and a logging feature to record errors.

Some parameters or limitations can also be incorporated to reduce unintended risks. The supplementary rule can be set as a best practice standard to determine the liability of designers, developers, operators, and customers (Rachum-Twaig, 2020). Dheu and De Bruyne (2023) proposed a new liability approach. They suggested implementing a ‘multi-faceted’ approach where consumer protection law, medical device laws, tort law, and strict liability should be jointly applied to any claim involving AI products. Smith and Fotheringham (2022) proposed a new tort known as ‘breach of statutory duty’ as mentioned in the Medicines and Medical Devices Act 2021 of the UK to regulate product liability in AI-enabled medical devices.

Custers et al. (2025) proposed a shared responsibility framework grounded in fiduciary duty. Fiduciary duties can be used to fill gaps in existing legislation. The AI has no distinguishable stages; it is the result of cooperation and co-creation. In such a situation, shared responsibility could be a solution, as no one has complete control over who is responsible for the fault or can prevent it. Shared responsibilities and fiduciary duties are to be implemented on the assumption that all stakeholders are liable unless proven otherwise. There is also a possibility of partial liability, reflecting the stakeholder’s share of responsibility. Since AI transforms the economy and boosts GDP growth, it is necessary to involve all stakeholders through equitable governance, alongside updated laws. The governance should address infrastructural gaps, algorithmic bias, and privacy violations (Tonieva et al., 2025). Inequalities and bias in algorithmic decision-making can be mitigated through human-centric, responsible AI that respects privacy, governance, and oversight principles. It should interact with humans while respecting their cognitive capacities to achieve a sustainable AI society (Ozmen Garibay et al., 2023).

In introducing AI regulation, it is proposed to avoid technology colonization and close the technology-related disparity. To achieve this, AI-related regulation should include five core principles: data exchange; autonomy; protection of values; accountability; and environmental sustainability. Successful implementation requires core infrastructure and coordination across sectors and borders (Morley et al., 2025). There is also a suggestion to adopt a technology-neutral regulatory framework. Failure to develop technology-neutral regulation will lead to underachievement in regulating AI technology, thereby affecting trust (Cordella and Gualdi, 2024).

The literature review showed that AI discussion is broad, and there is little on AI product liability. The coverage focuses mainly on responsible AI, AI ethics, copyright, and data privacy. The coverage on product liability is limited, and they discussed the application of tort liability, strict liability, or strict product liability to AI products or AI-enabled products in a limited context. There was little literature on EU and Canadian laws on AI product liability.

## 3 Methodology

To achieve the research aim, the researcher used a systematic literature review, legal doctrinal research, and comparative legal method. A systematic review of the literature was conducted to collect relevant research materials, assess and analyze them, and identify the research themes covered and the gaps in the literature. The researcher followed the inclusion and exclusion criteria in collecting the literature (Shahzad et al., 2025). The stage one search included the keywords or combinations of “product liability,” “artificial intelligence,” “law,” “EU,” and “Canada.” The research yielded 178 publications in the Scopus database. In the second stage, the search applied restrictions to articles in English and refined the keywords to “product liability,” “artificial intelligence,” “law,” “privacy,” “ethics,” and “AI Act.” This produced 37 journal articles, of which 20 were related to artificial intelligence, 5 to law, 5 to privacy, and the rest to humans. None of the research materials were related to product liability. Since there were no research materials on law and product liability in relation to AI, the EBSCO Connect database was searched in stage three. There were 117 retrieved. The abstracts were read, and none were relevant to the current research. The search was refined to include product safety, yielding 2 articles. In the final stage of the search, Google Scholar was used to gather additional research on the topic. All relevant articles were reviewed and analyzed for inclusion in the research (Magabaleh et al., 2024; Sarabdeen, 2024).

To conduct legal content analysis, the researcher searched relevant laws and regulations on product liability, artificial intelligence, tort liability, strict liability, and privacy (Hutchinson and Duncan, 2012; Alhussein et al., 2023). This search helped to understand the relevant law and evaluate the laws and regulations to answer the research aim. Some relevant cases were analyzed to examine liability for AI products and the parties liable for defective AI products and services. The research also used comparative analysis. Comparative research can offer a broader theoretical perspective for effective AI regulation. The regulation can incorporate liability along with the governance principle. It compared the EU Product Liability Directives to Canadian law. In this context, the PLD, AIA of the EU, and negligent liability in Canada have been compared. Since EU directives are considered comprehensive in regulating AI, and Canada follows the EU approach to AI regulation, comparing them with the relevant EU directives will help suggest improvements to the Canadian law.

**Figure 1:**
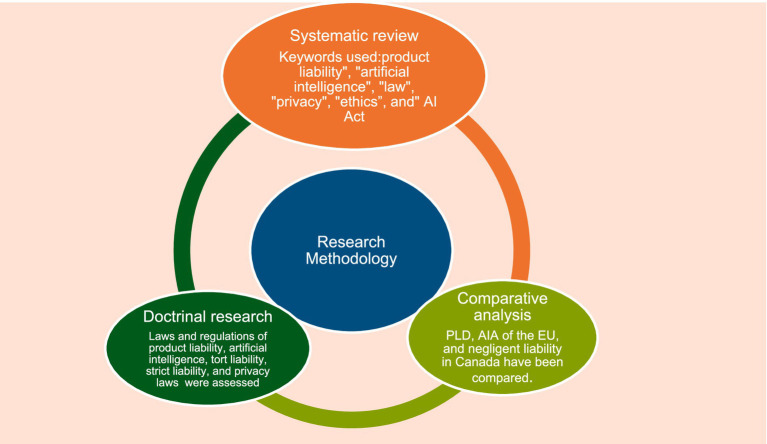
Research methodology.

All three methodologies can help to achieve the research objectives (Tanoamchard and Ceienwattanasook, 2024). Synthesizing and analyzing the literature helps to find the theme and the gap in the literature. Doctrinal research can assess the availability of laws regulating AI products and their liabilities (Greene et al., 1989). The comparative analysis will highlight the law’s strengths and weaknesses and can help propose amendments to achieve an effective liability regime. The combined approach helps expand understanding of applicable law in AI product liability. Figure 1 explains the research method used in the research.

## 4 Legal analysis

### 4.1 Liability regime-strict liability or negligence

If there is any harm caused by an AI product or an enabled product, a claimant has an option to sue the developer or manufacturer, or related parties under strict liability or negligent liability. Strict liability was introduced to protect the consumers. In a products liability claim, consumers are required to prove the product, the injury, and the causation between the loss and the product that caused it, unlike in a negligence claim. Under negligence, a claimant is required to prove duty of care, standard of care, breach of the standard of care, damage, and causation. Generally, consumers lack information about the risks associated with AI products. The purchase decision is made without understanding the product’s risks. Some countries impose strict liability on manufacturers to improve product safety features. Observable risks allow consumers to assess product risks and prices and make informed purchase decisions (Shavell, 2004). Since the risks in AI products are not easily observable, strict liability on the manufacturers may be a better option.

Additionally, a lack of knowledge and the cost to consumers may prevent them from reading the liability disclaimers. Any disadvantageous contractual clauses in consumer contracts may not be read or understood by them (Landes and Posner, 1985). In this scenario, strict liability can be a better option if the consumer is harmed. Some product risks cannot be controlled even after taking measures to reduce risks. The law tends to hold manufacturers strictly liable for failing to improve the production and sale of risky products (Geistfeld, 2021). If the manufacturer is aware of the product’s risk, strict liability could be used rather than negligence (Landes and Posner, 1985). An important factor in choosing between strict liability and negligence is assessing the party’s capacity to reduce harm through their activities. For example, strict liability for high-risk AI systems may be warranted given their opaque nature and post-deployment challenges. Strict liability can resolve the difficulties in establishing liability for AI defects (Irene and Andersen, 2025). However, Wendehorst (2020) suggested adopting strict liability only for physical risk, not for economic or social risks. This narrow application of strict liability can control the risks caused by AI products while also encouraging continuous development.

For highly dangerous products, if consumers are well informed about the danger, they can avoid buying the product or use it less, and manufacturers can be relieved of liability. Manufacturers can be exempted from liability only if consumers are accurately and sufficiently informed, and the risk is apparent or reducing the risk is difficult. Strict liability is imposed on inherently dangerous activities to ensure maximum care in production and reduce the use of the product to a socially optimal amount. Manufacturing defects or failures, and risky products, could lead to a strict liability claim (Friehe et al., 2022).

However, there may be no liability if the harm is caused by an identifiable risk, an occasional accident, wear and tear, or misuse of the product. The producers can take precautions in the manufacturing process. The precautions could include selecting high-quality materials, implementing a series of inspections, ensuring product consistency, maintaining safety, and fostering a quality culture. The safe design, comprehensive manuals, and warnings could also help control the risks associated with the product. Continuous research on product improvement and safety standards can be carried out as part of safety initiatives (Li and Visscher, 2020).

Under a negligence rule, manufacturers can be relieved of liability if they have in place safety measures. However, it is difficult to meet the safety requirements as it is difficult to define safety requirements in AI products, unlike in other products. The other difficulty in bringing a negligence action is proving causation. Proving causation in AI product liability cases will be difficult. If the defective products do not cause harm, no cause of action could be established (*Ide v ATB Sales Ltd* [2008] EWCA Civ 424). Therefore, proving causation on a balance of probability basis will be challenging in a tort liability claim if loss is caused by multiple negligent and non-negligent causes (*Wilsher v Essex Area Health Authority* [1988] AC 1074).

In strict liability cases, the victim’s burden of proof is lower; they need only establish the defective product, the harm, and causation (Smith and Fotheringham, 2022). There is no need to apportion fault to the designers, manufacturers, and others. In *Brookes v. Lyft Inc*., Lyft was held liable for the damage caused by its drivers, as it was in the best position to control the risk through its digital application. However, the claim cannot succeed if the chain of causation is broken or if the user is aware of the risks (*Howmet Ltd v Economy Devices Ltd*, [2016] EWCA Civ 847).

AI is *complex,* as multiple stakeholders are involved in the interdependence of AI components; thus, it is difficult to trace the source of a malfunction or attribute liability. The system is *opaque,* and it will be unclear which input resulted in the output that caused the injury. The AI is *autonomous*; it will be difficult to attribute to an actor, and when it acts completely autonomously, the action is unpredictable (Al-Dajeh et al., 2025). All these will make it difficult to establish causation between the AI product and the harm. It will also be difficult to establish a responsible party. Defining fault is difficult if the AI action cannot be reasonably anticipated. When choosing between fault-based negligence and strict liability, factors such as costs, the role of the injured party, the value of the products, and the seriousness of the risk should be considered. Strict liability will require close monitoring of system design, development, and implementation, and will help the claimant control costs in proving fault.

In controlling risk, insurance can play an integral role in hedging it. With insurance, the AI development will not be hampered by defective AI product claims (Roy, 2026). Besides, a new negligence standard for AI may be considered, under which an algorithm used in AI will be considered negligent if it causes frequent injuries compared to all actors in similar conduct. This new approach can set a baseline for AI negligence. This can incentivize developers to release safe AI products, thereby leading to product improvements (Diamantis, 2025).

**Figure 2:**
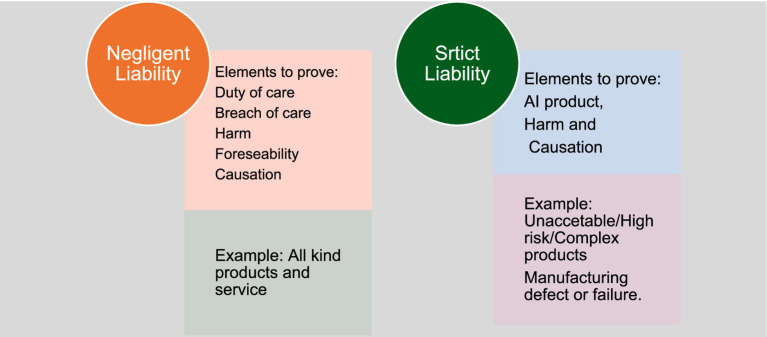
Negligent liability and strict liability.

Figure 2 provides elements for proving negligence and strict liability under the current legal system, along with examples of cases in which negligence and strict liability may be applied.

### 4.2 EU product liability framework

#### 4.2.1 The scope of the law

With the increased use of AI, the EU updated the Product Liability Directive (PLD) for defective products. The AIA regulation was passed on related issues (Dheu et al., 2022). The PLD extends product liability to cover all AI systems and AI-enabled goods, except open-source software. Open-source software is primarily excluded to ensure that research and innovation are not constrained (Articles 2(2) and 4(1) of the PLD). The updated PLD introduced the following:

1. Strict liability applies in limited cases, and the scope of covered products was expanded.
2. It replaced producers with various economic operators who can be sued for violations.
3. It introduced a liability of non-professional users of AI systems for violation of rights.
4. The requirement of disclosure mechanisms and rebuttable presumptions. The providers or deployers shoulder the burden of proof in technically complex cases (PLD Articles 9 and 10).
5. Damage includes medically recognized psychological harm and destruction or corruption of non-professional data; non-material loss is recognized to the extent compensable under national law (Art 6).
6. The limitation period for latent damage has been extended to 25 years.

According to the PLD, in a claim for defective products, the claimants need only show evidence of harm under Article 9. Noncompliance could lead to a presumption that the defendant has breached its refutable duty of care. PLD revision introduced a neutral definition of products covering digital, AI-related, and refurbished products. The amendment also enhanced the liability regime by extending coverage to AI-enabled products. PLD replaced “producer” with “manufacturer” to include software providers, digital service providers, and online marketplaces. The harm was extended to cover loss or corruption of data, psychological damage, and to allow the claimant access to relevant information.

The definitions of ‘claimant’ and ‘defendant’ are general, allowing a consumer to claim against a defendant who produces or is responsible for a defective AI system or output. Article 10 of PDL requires proof of the “defectiveness of the product, the damage suffered and the causal link between that defectiveness and that damage.” Since establishing the defect of an AI system is difficult for consumers, Article 10 includes a rebuttable presumption of causation. The presumption concerns the causal relationship between the harm caused, noncompliance, and the loss due to the defect. The presumption is only available if the court considers that it is difficult to prove a causal link.

The AIA classifies the risk and imposes different requirements for different types of AI systems. It provides safety-oriented rules, the establishment of a quality management system, registration, documents, cooperation, human oversight mechanisms, and a post-market monitoring system. According to the AIA risk categorization, “unacceptable risk” products are very risky and are prohibited from marketing. High-risk systems require *ex-ante* conformity assessments of safety, supervision, and related areas. According to Chapter V, high-risk AI systems and products must comply with the regulation’s requirements and be approved by the authority. Failure may lead to liability. *Limited-risk* AI systems may be marketed provided that manufacturers comply with the transparency requirement and that users are informed of their use of AI. Breach of the requirement may expose the party to tort liability.

Strict liability is said to be necessary for serious, but society-necessary, products. The PLD reduced the plaintiff’s burden by introducing a rebuttable presumption of liability in certain cases. The AIA proposed resolution and wider comprehensive civil liability options for AI product liability (Chamberlain, 2023). PLD and AIA should be read together to decide the liability of defective AI. PLD and AIA complement and reinforce one another.

#### 4.2.2 Limitations of laws in the EU and effective AI regulatory tools

AIA’s three-tier classification: standard, open licensed, and systemically risky, may fail to capture the peculiarities of various downstream applications (Novelli et al., 2024). Meeting the requirements of the law in the development of Generative AI will be challenging, as they do not have a specific purpose before adaptation (Bommasani et al., 2022). However, Xiao et al. (2022) suggest that combining conventional AI fault and defectiveness criteria with new monitoring methods can make AI more effective and help avoid liability.

AIA does not provide a redress mechanism; a claimant should refer to the PLD or other national laws for effective remedies. PLD and AIA combined can prove the link and fault of the output that caused the harm. However, there exist some differences. On disclosure of evidence, PLD requires disclosure of evidence of AI systems. On the contrary, AIA requires record-keeping for high-risk systems only (Article 12, AIA; Hacker, 2023). The types of documents to be disclosed are clear in PLD; however, the AIA does not mention the required documents. To meet the evidential requirement, records of training, testing, model evaluation outcomes, and legal compliance should be retained. AIA requires ongoing performance records. These records should also be preserved. The report should include incidents, incident descriptions with timestamps, logs, the standard followed, and a third-party audit (Falco et al., 2021). The train data disclosure should also be well documented, including the objective of data collection, data selection, and other relevant details (Bender et al., 2021; Jo and Gebru, 2020). On transparency, prior disclosure to consumers of the model they engaged, and its adverse effects can effectively meet the AIA mandate on notification, even though the law is vague on the notification requirement (Ziosi et al., 2023). Identifying potential risks and sharing them with users could preserve the desired evidence and foster transparency. Additionally, alliances with credible third-party and auditing agencies could help collect information on adverse effects.

According to the AIA, AI is a machine-based system that could generate output. The output can take the form of a prediction, decision, recommendation, or content that may impact individuals. If the products are listed in the Annexes of the AIA, the provider must conduct a conformity assessment. The conformity assessment may assess the number of AI systems likely to cause harm, the extent of the harm, the vulnerability of individuals caused by the AI system, the irreversible effects, and the extent to which the law provides remedies. The AIA requires disclosure of information about AI use, but it is unclear to what extent disclosure is required.

The PLD states that any natural person is entitled to damages if he suffers death, physical, psychological, or property harm. The law considered possible changes and upgrades to the AI system from the product’s initial launch through the claim stage. The definition also covers changes to a movable product that are eventually integrated as another movable or unmovable object. However, a claim for injuries caused by defective products can be awarded only if causation is established or the national courts find that the claim falls within a rebuttable presumption.

Though PLD explains liability, the AIA lacks specificity and does not elaborate on the liability. The parties need to review PLD and the relevant local legislation to understand the liabilities. The other challenge is that the AI system may not be covered under the PLD, as it is not a product but rather could be considered a service.

Proving defects could be an issue, too. It may be necessary to show a reasonable expectation that could lead to disputes over expected safety standards, as well as the foreseeability of misuse. This could shift liability from no-fault to fault-based. The developer or producer may use a latent defect and a state-of-the-art defense to escape liability. Regarding later defects, if they show the defect did not exist at the time of sale or circulation, the producer will have no liability to the developer. However, it will be difficult for a consumer to prove otherwise, as it involves complex software and untraceable AI decisions. Regarding the state-of-the-art defense, it is also difficult to prove. Article 18 of the PLD allows member countries to derogate from the development of risk defense.

The analysis of the PLD and AIA on AI product liabilities showed that the law provides a liability mechanism for AI products or AI-enabled products. However, there is a need for clarity regarding AI-enabled services. To avoid confusion regarding the application of PLD and AIA, they could be codified into one comprehensive directive to address AI liability issues. All AI model providers should be required to follow transparency, accountability, and record-keeping obligations. They should also be covered with a disclosure mechanism and a rebuttable presumption, as they have all the necessary information required by law. The law should provide details on the responsible parties and the ways to rebut the presumption.

To introduce an exemption from liability, the AI model’s classification should be taken into account. AI risk scenarios, classified by AI model type, would help clarify the liability of various stakeholders (Novelli et al., 2024). A risk-based approach is considered proportionate, and liability could be imposed in proportion to the risk. According to the AIA classification of risks, unacceptable risk is the highest level in the hierarchy, followed by high, limited, and minimal. The highest risk will require restrictive rules, such as prohibition, and less restrictive rules will be applied to the minimal risk category (Gasiola, 2025). The risk can be assessed based on the seriousness of the risk to the parties, the likelihood of injury even with safety measures in place, and the certainty and clarity of the law. Products that pose unacceptable risk should be prohibited (Hanif et al., 2024). Strict liability is suitable for regulating high-risk products, as they have the potential to cause harm or damage beyond what is reasonable for the AI product. The limited- and minimal-risk products will require transparency and accountability. Tort liability may be suitable to control liability.

In an AI product, liability allocation is crucial because multiple parties are involved in its development. A producer or any party responsible for the product can be held primarily liable for injuries, since safety and liability must be treated in parallel. Deployers may also be held liable for decisions on when and how to deploy the AI product, as they receive economic benefits. Further, they influence the level of exposure to risks. The backend operator could also be entirely responsible for defining the feature, providing updates, and backup support. They should also be held proportionately liable for their contribution to the injury, as they have control over the subsystem (Wendehorst, 2020). Additionally, the automatic presumption of AI systems under Annex III may be re-examined to authorize the Commission to adjust the risk categories and apply a rebuttable presumption.

### 4.3 AI product liability in Canada

#### 4.3.1 The scope of the law

##### 4.3.1.1 Negligence vs. strict liability

In the fault-based claim under the current Canadian law, the claimant should establish the duty of care, standard of care, breach of the standard of care, damage, and causation. Proving all these requirements for a defective AI system could be difficult for a claimant. Due to the evidential difficulties of proving fault, a strict liability was suggested as it is currently applied to harms caused by dangerous animals. Some argue that strict liability is also ineffective, as it could expose innocent parties in the AI supply chain to liability. Currently, the Product liability suits in Canada are mainly brought under negligence. In the common law provinces of Canada, if a claimant would like to file a case under a products liability action in tort, he needs to establish the following elements:

1. The product had a defect traceable to its manufacture, design, or warnings or instructions.
2. The manufacturer was negligent as regards this defect.
3. There is a causal connection between the manufacturer’s negligence and the damages suffered.
4. Damages are such that they give rise to a claim for compensation in law.

In a failure-to-warn case involving the use of a manufacturer’s product, two questions should be asked by the courts: did the manufacturer have a duty to warn? Moreover, was the duty breached? On the question of breach of duty, the courts focused on the reasonableness of the defendant’s conduct rather than on what an ordinary prudent person would do in similar circumstances, to avoid the floodgates of litigation. Thus, the courts are more concerned about the specific defendant at bar than the general duty of care.

The case of *Farro v. Nutone Electrical Ltd. (1990), 40 O.A.C. 233 (CA)* and *R. v. Leblanc, 1989 CanLII 56 (SCC), [1989] 1 SCR 1583* are examples of products liability cases where the Canadian courts should have considered strict liability where reasonable care is unnecessary; on the contrary, the courts applied negligence to avoid unjust results. In *LeBlanc*, the plaintiff was badly injured when a beer bottle shattered unexpectedly in his hand. In *Farro*, an overheated fan caused a fire that resulted in smoke damage to the house and its contents. The appellate courts in both cases linked the defect and injury to negligence, not strict liability. However, they failed to explain the defects and the reasonable care the defendant took with respect to their products. (Boivin, 1995).

According to Boivin (1993), in strict liability, the reasonableness of the manufacturer’s conduct is irrelevant, unlike negligence liability. Negligence considers the conduct of the manufacturer to be materially important. In strict liability, the causation of the product and damage to the plaintiff is crucial. When negligence is used to assess a product’s defectiveness, three issues regarding manufacturing conduct should be considered: the product’s manufacturing, its design, and adequate warnings. It is said that proving a manufacturing defect would be simple, as it requires a comparison with a similar product that caused injury to the claimant. However, proving defects in the warning will be harder. It is contented that defining negligence and strict liability is challenging, and that judges in Canada have used these terms gratuitously without differentiating.

The 1979 *Report on Products Liability* of the Ontario Law Reform Commission recommended strict tort liability for injury caused by defective products. However, the Commission did not elaborate on the meaning of the standard. Strict liability is used to contrast with the need to prove negligence to succeed. It also failed to elaborate on the standard of negligence. The courts in the USA and Canada sought to combine elements of negligence and strict liability to determine product liability. The courts consider the manufacturer’s foreseeability of the danger to decide on the duty to warn. If there is a duty to warn, they focus on the product’s defective nature and the adequacy of the warning. When there is a failure of warning, the court does not proceed with further investigation and holds the manufacturers liable. Here, there are no distinctions between negligence and strict liability. Boivin (1993) argued that adopting strict liability will not be an answer to design defects and warnings. Establishing correct tests and criteria for liability without fault will not be easy. However, Boivin (1995) proposed strict liability for manufacturing defects if the plaintiff demonstrates that the product is dangerously different from the original intended design, the manufacturer is responsible for the defect, and the injury was caused by the defective product design.

##### 4.3.1.2 Defect and causation tests

In proving defect and causation, the Canadian courts have moved from the reasonable man standard to the reasonable person test. Nonetheless, the standard is the same: an ordinary prudent person. It is an objective test and can vary depending on the defendant group (e.g., professional, children; Hutchinson, 2023). An objective standard is used as a tool that is based on a notional ordinary person to assess the plaintiff’s reaction. The application of this test depends on the decision maker’s construction of the model plaintiff (Lim, 2014). The Supreme Court of Canada in *Hollis v Dow Corning Corp* (1995 CanLII 55 (SCC), [1995] 4 SCR 634) used a subjective test. In *Hollis*, the manufacturer was sued for a failed breast implant, as there was an absence of warning of the risks of post-surgical complications. The possibility of rupture due to normal activity was given only after 2 years of the first operation, though the manufacturer knew the risk well in advance. The Supreme Court of Canada rejected an objective standard of causation. It adopted a subjective standard that the plaintiff herself would have used the product, but for the defendant’s breach. According to the court, the plaintiff should prove that the plaintiff would have acted differently had the warning been given. However, in product liability, it might be difficult to prove with certainty that the plaintiff would have modified the conduct but for the defendant’s negligence.

Many expressed concerns about relying exclusively on a test of idealized personhood as a one-size-fits-all approach (Lewans, 1996). A pragmatic approach to factual causation could allow the court to deviate from the presumption of causation on a case-by-case basis (Boivin, 1998). The court should have taken a pragmatic approach to factual causation, applying the subjective standard with the presumption that consumers follow the warnings provided by manufacturers. Factual causation in relation to failure to warn looks at two concepts, namely, injury causation, where the risk in defendant products and the damage suffered by the plaintiff are considered. Plaintiffs cannot succeed without establishing this on a balance of probability (*Buchan v. Ortho Pharmaceutical (Canada) Ltd*, (1986), 12 O.A.C. 361 (CA)).

In medicinal products, causation in product liability involves proving general causation, where it should be shown that the product is capable of causing injury to the public, and a specific causation that the substance caused injury to the plaintiff. Causation should be established on the balance of probabilities. In *Vadera v. Shaw*, the English Court of Appeal reconciled the balance of probabilities with the scientific standard of statistical significance or epidemiological evidence in the case of medicinal products (Goldberg, 2014).

Negligence and related personal law have been considered inadequate to claim compensation for injury caused by innovative products. Proof of negligence in innovative products is similar to the requirements of pharmaceutical litigation: proof of failure of product safety before the product enters the market and failure to warn of the risks associated with it. This requires expert examination, protocols, and trials that would pose problems for an ordinary plaintiff (Arbour, 2014).

##### 4.3.1.3 Recoverable heads of loss

Physical injuries and property damages are undisputed heads of recoverable harm. Psychiatric harm may be recoverable as part of personal injuries (Hutchinson, 2023). However, recognition of pure economic losses and so-called wrongful birth or life is still underdeveloped in Canada. To claim economic loss, the loss should be the result of negligent statements, and it should be consequential to personal injury or property damage. Whether pure economic loss is a recoverable head of damage is not entirely clear.

Though pure economic loss is recoverable, only losses in limited categories are allowed. In *Ontario Inc. v. Maple Leaf Foods Inc.* (2020 SCC 35 (CanLII), [2020] 3 SCR 504), the franchisees suffered losses due to MLF’s negligence. MLF was not party to the franchise agreement, though the Franchise agreement included MLF as the meat supplier. The 2-month delay in meat delivery to franchisees led to business closures. The Franchisee claimed against MLF for loss of future profits and a reduction in the capital value of the franchises. The Supreme Court found that the claim is for pure economic losses, not linked to personal or property damage, and therefore not recoverable. According to the Supreme Court, the parties lacked proximity that would have created a duty to avoid such losses. The MLF quality and fitness of their products to the consumers, not the franchisees, and the franchisees could have opted out of the exclusive supply agreement with MLF.

Hutchinson (2023) proposed a transformative approach to introduce a general rule allowing vulnerable parties to recover pure economic losses resulting from the defendant’s negligent actions. Such a vulnerable claimant could not have spread or insured such a loss; the defendant should have been held liable for the direct economic consequences.

In Arndt, the pregnant plaintiff was not warned of the impact of chickenpox on her unborn child. The plaintiff claimed that she would have terminated the pregnancy had she known the risk. She claimed for child-rearing costs and loss of income. The Supreme Court, applying the “modified objective” test, decided against the plaintiff. The court found that she would not have terminated the child since she wanted children and the risk of harm was small. The court, in its decision, failed to consider a substantial connection between the negligent defendant and the plaintiff’s harm, instead focusing on the plaintiff’s position. It also ignored that the defendant failed to provide reasonable and expected advice, thereby denying the plaintiff a choice. It is suggested that a new approach should be taken where a defendant should be made liable if there is a sufficiently real and substantial connection to the plaintiff’s harms (Hutchinson, 2023).

The liability of a general contractor who is not a party to the contract has been discussed in *Winnipeg Condominium Corporation No. 36 v. Bird Construction* (1995 CanLII 146 (SCC), [1995] 1 SCRSCR 85). The Supreme Court of Canada held that the general contractor should be liable for the cost of repairing defects in the building resulting from negligence in its construction. The SCC applied the Anns and Kamloops two-part test on economic loss, under which the contractor may be held liable if he reasonably foresees that negligent design or construction defects in a building are latent and that subsequent purchasers may suffer physical or property injury. If the defect is discovered before the injury, it may pose a real and substantial danger, and the contractor should be liable for the repair costs. SCC followed *Rivtow Marine Ltd. v. Washington Iron Works* to recognize economic loss to protect the bodily harm and property interests of the residents. The duty in tort serves to protect the bodily integrity and property interests of the building’s inhabitants.

##### 4.3.1.4 Allocation of loss among economic operators

When various parties are involved in a product injury case, one may want to look beyond superficial appearances of the injurer and the injured. This will allow us to investigate other parties and devices that played an important role in absorbing the cost of accidents. Loss allocation can be assigned “vertically and horizontally. Vertical loss assignment looks at various parties, while horizontal loss allocation looks at various sources of liability other than tort liability. This will be considered as loss-shifting to the loss-spreading function of tort law. This will help to alleviate the burden of adverse judgment and strategically spread the cost most fairly and economically (Fleming, 1966).

Economic loss could be recovered under five categories. They are statutory public authorities’ independent liability; negligent misrepresentation; negligent services; defective goods or structures; and Relational economic loss. Negligent misrepresentation cases in which professionals, such as surveyors and accountants, make representations that cause loss to the plaintiff who relies on them. The liability also extended, with some modification, to cover solicitors negligent in effecting the testator’s wishes (*Ross v Counters* [1977 R. No. 3876]).

For negligence in supplying defective goods or structures, following the Bird Construction decision, the owner of the defective product or building can seek damage to the property, reduction in value, or cost of repair. Negligent inspection by statutory public authorities may lead to liability in negligence, even though the builders were not found liable (*Kamloops v Nielsen* 1984 CanLII 21 (SCC), [1984] and *Canadian National Railway Co. v. Norsk Pacific Steamship Co.,* [1992] 1 SCR 1021, 91 D.L.R. (4th)).

Relational Economic Loss holds a defendant liable for negligent damages caused to a third party to his person or his property. Though the majority of the claim failed, the court in Norsk Pacific Steamship Co held that the claim was for pure economic loss, which lacked the proximity to create a duty of care. The court assessed the various policy considerations in rewarding economic loss of this type.

### 4.4 Limitations of laws in Canada and effective AI regulatory tools

The Canadian legal framework for AI product liability is primarily centered on tort liability. Proving negligence will be challenging in AI product-related liability, especially in collecting relevant evidence in technologically complex AI systems. In *Hollis*, the Canadian court defined the relationship between a manufacturer and a consumer as a relationship of reliance, in which the consumer has less knowledge of a product’s inherent dangers. As technology becomes more complex, proving becomes difficult for consumers, and, as such, they are entitled to proof facilitation. Applying these cases, one can argue that the AI system developer or related parties should provide available evidence to consumers who do not understand the complexity of the AI system, as stated under the EU directives.

Seamless development of the AI market requires legal certainty, and, as such, a comprehensive federal legislative framework is necessary in Canada. The future regulation should be in harmony with other international trading partners and regulations. For example, classification of AI products should follow the internal standard, unlike the demised AIDA definition, which tries to categorize deeming generative, general-purpose AI products as “high-risk.” Such harmonization will facilitate mutual recognition and enable Canadian companies to enter the international market.

Exclusion of the public sector should be avoided, as participation in its services is involuntary, and there is no alternative for citizens. Excluding the public sector from regulation left important stakeholders out of the regulatory landscape. Future laws should include all the sectors (Fraser and Dykema, 2026). It should address AI product liability and elaborate on critical elements of fault-based liability. The burden of proof should be reduced, as consumer fault-proofing could be burdensome given the complex nature of algorithms. It should incorporate a presumption of liability for complex AI systems. The liability regime should include personal remedies or recourse.

There should be a record-keeping requirement of high impact, similar to the high-risk requirement, in future legislation. There should be a provision regarding the disclosure of this information to assist victims of the AI system. Hence, the consumers or claimants can access the records to support their claim. This will be in line with the court’s opinion in *Hollis*, which held that consumers may provide evidence to support their claims.

Controlling risk could minimize product liability claims, providing economic benefits for AI developers. As to who is liable for the AI-caused harms, it may be possible to apply the principle that the least-cost avoider should be allocated the means to mitigate the risks. The future law should sufficiently delineate the roles and responsibilities of the various parties in AI-related claims, thereby reducing liability-related uncertainty. Many organizations can be involved in design, development, or deployment, and not assigning roles and responsibilities opens the way to bring many parties into a lawsuit without knowing who is to be blamed for the harm. The EU PLD defines and explains the roles and liabilities, including those of economic operators. Economic operators can include manufacturers of products or components, service providers, and distributors. The economic operators can be held liable for harm caused by defective output and failure, depending on their involvement. Future legislation will impose joint liability on developers and deployers.

Future legislation should pay special attention to risk categorization and assessment to determine the type of risks to which the responsible person can be assigned for safety assessments, measures, and audits. By doing so, the liabilities of various parties can be specified. Further, compliance obligations should be added, and any violation of noncompliance should lead to liability. They should include consideration of possible changes and upgrades to the AI system from the product’s initial launch through the claim stage, and cover possible changes to a movable product that is eventually integrated as another movable or unmovable object, as provided in PLD.

Future legislation should also avoid a constitutional challenge to the division of legislative power. AIDA proposed legislation on AI-related trade, which the federal government lacks the power to regulate. The new law should ensure that the AI commissioner is an independent and credible authority, similar to the competition commissioner and the privacy commissioner, and answerable to parliament, not to the minister.

One of the issues raised as problematic under AIDA is the regulation that includes basic research and development on AI. It is suggested that the future AI regulation should place the research on low-risk categorization (Fraser and Dykema, 2026).

The EU has a better liability framework than the Canadian regulatory regime. PLD covers liability rules; AIA provides the legal framework for those rules to apply; and it serves as a moderator that benefits all businesses and consumers. However, in Canada, any AI product liability claim can be pursued under existing laws on tort, extracontractual civil liability, privacy, and human rights violations. However, proving the fault of complex AI systems with many parties in their supply chain can be challenging.

### 4.5 Comparison of regulatory frameworks for AI in Canada and the EU

The comparative analysis provides insights into how liability with governance shapes the market. It effectively integrates technical governance with liability alongside certification, auditing requirements, and ethical principles for responsible AI (Floridi et al., 2018). The liability regime alone, which requires proof of defect, causation, foreseeability, and injury, is difficult to establish for AI products, as they involve opaque, continuously updated systems and distributed responsibilities.

It also revealed that the Canadian and European regulatory approaches are distinct. The European regulatory system provided a harmonized, liability-modernized, and risk-based hybrid governance. The Canadian regulatory system is underdeveloped in AI governance, though it prides itself on a flexible liability framework. The identification of these differences can lead to broader discussions on designing a balanced and effective regulatory framework that promotes innovation and addresses consumer protection. The comparison of the regulatory frameworks is shown in Table 1.

**Table 1:** Comparison of the regulatory frameworks in Canada and the EU.

Design, deployment, governance issues	Canada	European union	Regulatory implications
Doctrinal philosophy	Relies on existing laws that are principles-oriented and fragmented, as well as sectoral-oriented (Scassa, 2023).	Harmonized, risk-based, and comprehensive regulation (European Commission, 2021)	EU’s ex ante model encourages governance compliance in AI design and development; Canada lacks comprehensive AI governance regulation, though the current framework can be applied to AI design, deployment, and implementation.
Ai-centric laws	With the demise of the Artificial Intelligence and Data Act (AIDA) under Bill C-27, there is currently no legislation or law on this aspect.	The AI Act and the revised Product Liability Regulation impose responsibilities that depend on the risks an AI system may pose.	EU regulation imposes compliance by design. There is no specific regulatory requirement in Canada regarding the compliance of AI systems, their design, or their deployment, except for those imposed by tort law.
Liability	Fault and compensation (ex post)	Covered both liability (ex post) and governance (ex ante) (Wendehorst, 2020)	In the EU, the use of hybrid methods shapes the liability governance framework, encouraging safer, risk-aware design and deployment. The liability regime in Canada needs improvement to incentivize a safer design and application.
Ai product liability framework	Limited application of negligence and product liability principles.	Regulated through the revised Product Liability Directive and AI Act	EU adapts liability to address AI’s complexity, opacity, and autonomy; Canada follows existing doctrines and may not cover AI’s complexity.
Causation and burden of proof	Causation and burden of proof follow the tort requirement.	The Product Liability Directive modified the causation requirements and provided the possibility to shift the burden of proof in complex AI products.	The EU modernized product liability law to reduce evidentiary barriers and enforcement difficulties—no new developments in Canada in this regard.
Product risk-categorization	Following AIDA’s demise, risks are assessed under common law negligence and product liability principles.	The AI Act clearly classified risks into distinct categories.	In EU governance and compliance, the approach differs depending on the AI Act categorization. Canada follows common law requirements.
Accountability	It is through common law liability.	Accountability applied through the revised Product Liability Directive and AI Act (Veale and Borgesius, 2021)	Accountability is internalized in the EU through documentation, traceability, human oversight, and auditability, whereas Canada introduces it through a general liability framework. There is no legislation on AI-specific accountability.
Transparency	The privacy law may apply to AI products and systems.	The AI Act introduced compulsory transparency obligations for certain AI products.	It impacts explainability and AI system design.
Standards and certification	In the absence of regulatory interference, reliance is placed on voluntary standards and guidelines.	Law and the regulation harmonized standards.	In the EU, laws and regulations introduced a structured compliance ecosystem, unlike in Canada.
Regulatory balance	A flexible regulatory environment that may be innovation-friendly.	It is a safety-oriented and rights-based approach.	The EU tries to balance between rights, trust, risk mitigation, and innovation. Canada lacks a comprehensive legal framework.
Governance model	Flexible, disintegrated, and adaptive	Centralized, integrated, lifecycle-based.	In the EU, the laws and regulations provide broader governance tools (Floridi et al., 2018), and Canadian AI governance is mainly self-regulatory.

## 5 Conclusion

The introduction of AI and AI-enabled products raised questions about the suitability of existing laws to regulate them. Proof of fault under tort law requires a duty of care, a breach of that duty, injury, foreseeability of the injury, and causation. In an AI environment, proving fault is challenging; as such, the EU directive includes strict liability and a rebuttable presumption of causation. The presumption concerns the causal relationship between the harm caused, noncompliance, and the loss due to the defect. This could protect consumers and encourage the designers, developers, manufacturers, and others to consider safety in all stages of AI system development. Though the EU’s product liability regime for AI products is better than in other countries, it still has some issues that need to be addressed. For example, regulations should clarify the application of the directives to AI services, the burden of proof, defenses, and recoverable damages. To avoid confusion among the three existing directives, they can be codified into a single comprehensive directive to address AI liability issues.

The Canadian government drafted AIDA to address issues related to AI, and some provisions address product liability. The draft was intended to follow the EU’s approach to regulating AI, but it died before it could be passed. In its current form, consumers need to prove the AI system’s fault in causing harm under the law of negligence. The requirement for consumers to provide proof of fault could be burdensome, given the complex nature of algorithms. Any future legislation should spell out the liability regime and include personal remedies or recourse. The roles and liabilities of the parties in the AI ecosystem should be clarified to reduce uncertainty about the responsible party for the damage. The future law should apply to all sectors.

The research has a few limitations. In terms of research methodology, a systematic literature review, legal doctrinal research, and comparative legal method were used to achieve the research objective. Using this method, the research reviewed the available literature to identify gaps, identified laws and regulations, and assessed their suitability for regulating AI product liability. Future research can employ additional qualitative and quantitative methods to investigate product liability across various contexts. The other limitation concerns the scope of the research. The research focused only on the directives and laws of the EU and Canada, and the findings may not generalize to countries with similar systems. However, future research may expand the coverage. Another limitation is that the research only assesses the laws in relation to AI products, and it does not assess the laws that apply to other products and services.

## Data Availability

The original contributions presented in the study are included in the article/supplementary material, further inquiries can be directed to the corresponding author.
